# Governance of non-state-owned shareholders and corporate capital structure decision: A mechanism test from the opportunistic behavior of management

**DOI:** 10.1371/journal.pone.0281120

**Published:** 2023-01-27

**Authors:** Jiao Wang, Yaoyao Hu, Feimei Liao, Shulin Xu

**Affiliations:** 1 School of Economics, Guangzhou College of Commerce, Guangzhou, P. R. China; 2 School of Finance, Jiangxi Normal University, Nanchang, P. R. China; University of Baltistan, PAKISTAN

## Abstract

It is an important measure in the reform of state-owned enterprises to improve the efficiency of capital operation by introducing non-state-owned shareholders. This paper explores the impact of non-state shareholder governance on capital structure decision-making by using the data of 2008–2021 A-share state-owned listed companies from the perspective of the speed and deviation of capital structure adjustment. The results reveal that only non-state shareholding has no significant impact on the capital structure adjustment of a company. However, the appointment of senior management by non-state shareholders can speed up the capital structure adjustment and lower the degree of capital structure deviation. Moreover, when the capital structure goes down, the appointment of non-state-owned shareholders plays a larger role in accelerating the capital structure adjustment, which makes the deviation from the actual capital structure and the target capital structure smaller. Further research shows that the above relationship between non-state shareholder governance and the optimization and adjustment of the capital structure only exists in local SOEs and competitive SOEs. In addition, the path test found that non-state shareholder governance affects the dynamic adjustment of capital structure by reducing opportunism behavior of management rather than by financing constraints.

## 1. Introduction

In December 2016, the Central Economic Work Conference put forward, “In terms of deleveraging, in order to control the total leverage ratio, it is important to reduce the leverage ratio of enterprises.” In August 2017, Premier Li Keqiang presided and convened an executive meeting of the State Council and pointed out that “at present, it is necessary to seize the opportunity for central enterprises to turn their profits around to upward, and to take leverage reduction of state-owned enterprises as the top priority of deleveraging.” In terms of internal mechanism, the reasonable measure of the leverage ratio is the asset-liability ratio, which is the ratio of total liabilities to total assets. Correspondingly, the capital structure is often characterized by the relationship between debt and equity [1]. According to the accounting identity, “asset equals to debt plus equity”, it is obvious that the leverage ratio and the capital structure seem to be two sides of a coin [2], “deleveraging” in essence, is the downward adjustment of the capital structure of the entity, however, the downward adjustment is not to reduce the leverage ratio of the physical enterprise to zero, but to adjust its micro-debt ratio to the risk threshold for effective prevention and control, so as to avoid financial crisis caused by excessive debt [3]. Due to the constant development and change of a company’s internal and external environment, competitive pattern and its own financial operating status, the threshold of the optimal capital structure of the company is determined to be dynamically adjusted continuously [4–5] to adapt to the changes of its internal and external environment. Therefore, from a dynamic perspective, the essence of deleveraging is the dynamic adjustment process of capital structure [2, 6].

However, an outstanding question is whether the governance of non-state shareholders affects the dynamic adjustment of the capital structure? Dynamic capital structure adjustment refers to the fact that firms aiming for value maximization have an incentive to recapitalize when their capital deviates from the target level [7]. The company’s adjustment of capital structure is inevitable due to the problems of transaction costs and information asymmetry on both sides of the capital market [8].For modern state-owned enterprises (SOEs), capital structure decision is one of the most important financial decision-making. In the course of business development, the capital structure of SOEs often deviates from the Pareto optimal level due to various interference factors. Under such circumstances, how to optimize the capital structure by constructing an effective corporate governance mechanism is at the core of modern corporate financial decision-making [4, 9]. In the external environment that affects the decision-making of the capital structure of SOEs, the study of institutional background has attracted more and more attention from scholars and has become a research hotspot in the field of dynamic capital structure. Existing scholars have discussed the institutional environment such as product market competition, legal environment, media coverage, capital market development, and macro-policy uncertainty, and have produced fruitful results, thereby expanding the capital structure theory [10–12]. In addition to the above institutional factors, few literatures examine how non-state shareholder governance affects corporate capital structure decisions at the macro-institutional level. Therefore, based on the previous research, this paper attempts to explore the relationship between the institutional arrangement that non-state-owned shareholders participate in the governance of SOEs and the decision-making on capital structure, and to discover whether and how non-state-owned shareholders’ governance affects the capital structure decisions-making in SOEs.

This study expands the existing literature as follows. Firstly, from the new perspective of non-state-owned shareholders’ participation in the governance of state-owned enterprises, this paper discusses the influence of non-state-owned shareholders’ governance on the speed of capital structure adjustment and the degree of capital structure deviation, which enriches the research on the influencing factors of capital structure decision-making and helps us understand the motivation and formation mechanism of capital structure decision-making of state-owned enterprises under the mixed ownership reform. Secondly, given that capital structure decision-making is an important part of financial decision-making, this paper gives a deep insight of the capital structure decision-making behavior and its causes. This article examines whether non-state-owned shareholder’ governance affects capital structure through opportunistic behavior of management or financing constraints, which helps to evaluate the explanatory power of principal-agent theory and financing constraint theory for capital structure decision-making. Thirdly, through the heterogeneity test of central state-owned enterprises and local state-owned enterprises, monopolistic state-owned enterprises and competitive state-owned enterprises, to find out which type of enterprise’s non-state-owned shareholders have more obvious influence on the dynamic adjustment of capital structure. Finally, this paper enriches the research on the economic consequences of non-state-owned shareholders’ governance from the perspective of dynamic adjustment of capital structure, which is conducive to understand the relationship between the non-state-owned shareholding ratio and the top executive appointment in firms with mixed-ownership reform and company value. Therefore, it provides a certain policy reference value for non-state shareholder governance. Furthermore, this article also finds an enlightening significance for the decision-making behavior of capital structure in mixed-ownership reform enterprises.

The remainder of this article is organized as follows: Section 2 presents theoretical analysis and research hypothesis. Section 3 proposes research design. Section 4 analyses empirical results. Section 5 summarizes conclusion and implication.

## 2. Theoretical analysis and research hypothesis

### 2.1 Governance of non-state-owned shareholders and capital structure decision-making

“Owners’ absence” in state-owned enterprises leads to a lack of effective supervision on senior executives, which increases the opportunistic behavior of senior executives and results in serious agency problems [13]. Top executives in SOEs often have the dual status which are associate officials and managers. In order to obtain high salaries and get political promotions, they may embellish their personal performance by building a personal “empire”, manipulating surpluses, and borrowing heavily. Therefore, external constraints and incentive mechanisms are required to correct the managerial agency behavior of top executives in SOEs. Ownership structure can affect decision-making in the process of corporate governance [14]. Specifically, shareholders who own equity can influence participating and voting in the general meeting of shareholders to impact the micro-behaviors of corporate executives. Therefore, the introduction of non-state capital and the participation of non-state shareholders in the governance of SOEs by the mixed-ownership reform is an expedient to improve the level of corporate governance [15]. The mixed-ownership reform of state-owned enterprises reappeared in the political posture of the top-level design. This introduced non-state-owned capital and assigned senior executives to actively participate in the governance of state-owned enterprises, which then, to a certain extent, alleviate the opportunistic behavior, such as the “owner absence” and “internal control”, thereby improving the quality of corporate governance in state-owned enterprises [16]. Moreover, the “profit-seeking nature” of non-state-owned capital make non-state-owned shareholders to supervise the management of state-owned enterprises, thereby improving corporate performance and increasing corporate value.

It has become a consensus in the academic world of modern finance that the target capital structure exists in an enterprise [17]. The adjustment speed and deviation degree of the actual capital structure to the target capital structure of a firm are important contents of the capital structure decision-making, and they are good measurements to the quality of the capital structure decision-making. The shorter the adjustment speed and the smaller the deviation degree, the more conducive to enhancing corporate value, the more efficient capital structure decisions [18]. According to Flannery and Rangan [17], since the operating environment of a firm is uncertain, its actual capital structure will deviate from its target capital structure. Also, environmental uncertainty will affect the speed of capital structure adjustment. The adjustment speed and the deviation degree will impact the value of the firm.

Wu et al. [19] find that the willingness to dynamically adjust the capital structure of top executives in state-owned enterprise will directly affect the adjustment process and adjustment speed of the capital structure. If SOEs have serious agency problems, even if the adjusting gain is greater than the adjusting cost, the executives may not make any adjustments to the capital structure. Studies also show that the management agency problem will affect the company’s cash flow distribution, and the higher the agency cost of a company, the slower the capital structure adjustment [3]. In the process of mixed-ownership reform of SOEs, the participation of non-state-owned shareholders in governance will effectively supervise and restrict the behavior of senior executives, thereby reducing agency costs and improving quality of governance, which reduces the adjusting cost of corporate capital structure [20]. Since the adjusting cost is an important factor that affect the speed of capital structure adjustment, under certain conditions, the smaller the adjustment cost and the faster the adjustment speed, the smaller the deviation between the actual capital structure and the target capital structure [21]. Therefore, non-state-owned shareholders can effectively influence corporate capital structure adjustment decisions by appointing senior executives to participate in SOEs’ governance. In addition, Zhang et al. [16] find that non-state-owned shareholder’ governance can improve the sensitivity of executive compensation performance; and Frank and Goyal [22] find that incentive compensation for executives is conducive to speed up the adjustment of capital structure. This also shows that through constraints and incentive effects, SOEs can reduce management opportunistic behavior, improve governance levels, and reduce adjusting costs, thereby affecting capital structure adjustment decisions. Based on the analysis, the following hypothesis (H1) is proposed.

**H1: *The higher the degree of governance of non-state-owned shareholders*, *the more able to reduce the opportunistic behavior of management*, *so that the speed of capital structure adjustment of state-owned enterprises is faster*, *and the degree of deviation from the target capital structure is smaller*.**

In the course of daily operations, the actual capital structure often deviates from the target capital structure due to a variety of internal and external factors, either higher than the target capital structure or lower than the target capital structure. When the actual capital structure is higher than the target capital structure, companies are prone to financial difficulties, and the marginal benefit of debt interest tax deduction is less than the marginal cost of the financial crisis. Therefore, rational SOEs’ executives will adjust the capital structure downward; vice versa, state-owned enterprise executives will adjust the capital structure upward [19]. In psychological research, Tversky and Kahneman [23] propose the prospect theory that people’s feelings about potential losses and the same amount of possible gains are often asymmetric. Moreover, people’s psychological fluctuations when they suffer losses are usually larger than those when they gain. In this case, the executives of SOEs are more sensitive to potential losses than potential gains, and they are more likely to avoid losses than to increase gains. Therefore, it is inevitable that in the process of capital structure adjustment, the effect of non-state-owned shareholder’s governance on the adjustment speed of capital structure will be impacted by such asymmetry. Memon et al. [24] show that the downward adjustment is more sensitive and timelier than upward adjustment. Consequently, in the process of dynamic adjustment of capital structure, SOEs’ senior executives are more likely to speed up the downward adjustment and reduce the degree of deviation due to the over-indebted enterprise’s capital structure, since under the pressure of high debt by the supervision and restraint of non-state-owned shareholders. Therefore, hypothesis 2 is proposed.

**H2: *Compared with the upward adjustment*, *the governance of non-state-owned shareholders will prompt the executives to accelerate the downward adjustment of the capital structure and reduce the deviation of the capital structure*.**

### 2.2 Regulating effects of government control levels and industry competition

Mixed-ownership reform introduces the non-state shareholders in state-owned enterprises, which can affect senior executives’ decision-making from equity checks and balances and corporate governance. This is mainly shown as the fact that the supervision and restriction from the non-state shareholders’ executives will impact the financial decision-making behavior of top executives, thus improving the internal governance mechanism in SOEs [16]. However, since there are many differences in SOEs, such as the levels of government control and market competition, the roles played by non-state-owned shareholders in corporate governance are also different, and their decision-making behavior must be affected by all levels of government [25]. Therefore, the decision-making behavior of capital structure among different types of state-owned enterprises is also various.

According to Chen et al. [26], this paper divides state-owned enterprises into central state-owned enterprises (CSOEs) and local state-owned enterprises (LSOEs) based on the level of government control. Because state-owned enterprises are controlled by different levels of government, their internal governance and decision-making behavior will undoubtedly be affected by different levels of government. The CSOEs have a very large market, can attract a high degree of attention among society and involve many stakeholders [27]. Obstacles from interest groups may impede supervision and governance effects on mixed-ownership reforms of CSOEs. The reasons are: First, central enterprises are in a vital strategic position in some important industries and core areas related to the national economy and people’s livelihood, and are therefore more under the control of the central government. This weakens the non-state-owned shareholders’ voice in the enterprise, which will inevitably affect non-state-owned shareholders to participate in the corporate governance. Second, the controlling shareholders of CSOEs are the central ministries and commissions. It is difficult for non-state-owned capital to form equity balance in CSOEs, thus limiting the alleviation of the agency problem in substance. Therefore, mixed ownership reform can hardly affect the financial decision-making behavior of senior executives in CSOEs. On the contrary, compared with CSOEs, LSOEs have less influence and the mixed-ownership reform are promoted more vigorously. Non-state-owned major shareholders are also more likely to enter the board of directors and have more rights to vote, so that non-state-owned shareholders can play a greater role in governance. The hypothesis 3 has been proposed as follow.

**H3: *Compared with CSOEs*, *the higher the degree of governance of LSOEs’ non-state-owned shareholders*, *the faster the speed of capital structure adjustment*, *and the smaller the degree of deviation from the target capital structure*.**

As mentioned above, the introduction of non-state-owned shareholder governance in state-owned enterprises can reduce the problem of managerial agency, thus affecting the decision-making behavior of executives on capital structure. However, this mechanism will be affected by a variety of factors. Since the levels of competition for various SOEs vary from industries, the mixed-ownership reform of state-owned enterprises may have different effects on capital structure decision-making behavior. On the one hand, monopolistic SOEs obtain high monopolized profits by their political and economic advantages, and it is often difficult for non-state-owned shareholders in such SOEs to participate in high-level governance through the appointment of senior executives. On the other hand, the monopolistic enterprises rely on the industry’s monopoly status to acquire competitive advantages. Even if private capital is introduced, allowing non-state-owned shareholders to participate in high-level governance, their enthusiasm for governance is difficult to exert. Moreover, those non-state-owned shareholders are more likely to keep a good relationship with those state-owned senior executives in order to earn the “reap where one has not sown” monopoly gains, thereby ignoring their roles of supervision. Therefore, compared to competitive SOEs, the governance role of non-state-owned shareholders in monopolistic SOEs may not be obvious.

Generally, the profits of competitive enterprises are mainly maintained by efficient business decisions. Out of their own interests, the introduced non-state-owned shareholders will pay more attention to the risk control in firms, including operating risks and financial risks. They have a strong motivation to monitor management and pay attention to the decision-making in top executives on operation and financing. The hypothesis 4 has been proposed as follow.

**H4: *Compared to monopolistic SOEs*, *the greater the degree of governance by non-state shareholders in competitive SOEs*, *the faster the capital structure adjustment and the smaller the deviation from the target capital structure*.**

## 3. Research design

### 3.1 sample and data

China’s shareholding reform was completed at the end of 2007, and then state-owned listed companies introduced non-state-owned capital one after another. Therefore, this paper selects 2008–2021 as the research sample. To ensure the validity of the data, we screened the initial sample according to the following criteria: (1) considering the particularity of the capital structure for financial industries, this paper eliminates such industries; (2) the sample excludes ST and PT listed companies, because the asset-liability ratio of such companies may be unstable and affect the research results; (3) and excludes negative assets, data with outliers and incomplete data, and finally we obtained 6005 company-year observations. The data in this paper come from the CSMAR database and are manually organized. To avoid the influence of outliers, a 1% double-tailed treatment is conducted on continuous variables.

### 3.2 Model design and variable definition

This paper follows Faulkender et al. [3] and Byoun [28], uses the speed of capital structure adjustment and the deviation degree between the actual capital structure and the target capital structure to reflect the decision-making on capital structure and constructs models to verify it. Firstly, we follow Faulkender et al. [3] to construct the target capital structure:

Lev*=α0+α1Size+α2Roa+α3Fixass+α4Revgro+α5Depre+α6Lev_med+α7Year+u (1)

In this formula, Lev* is the target capital structure of the enterprise at the end of the year, *Size* is the natural logarithm of the asset size at the end of the previous year; *Roa* is the return on total assets at the end of the previous year; *Fixass* is the level of tangible assets at the end of the previous year, which is represented as the sum of the inventory and fixed assets at the end of the previous year divided by total assets; *Revgro* is the growth rate of operating income at the end of last year; *Depre* means non-debt tax shield, which is represented by the ratio of fixed asset depreciation and total assets at the end of last year; *Lev*_*med* is expressed by the median of the capital structure of the enterprise in the same industry in the previous year; *Year* is an annual dummy variable, *u* represents the special non-observed effect of the enterprise. The target capital structure is the fitting value of the asset-liability ratio calculated based on the regression of the model (1), which is recorded as Lev*.

Secondly, according to the models used by Flannery and Rangan [17] and Byun [28], the capital structure adjustment speed model is constructed as follows:

$$Lev=(1‐\delta )\;LagLev+\delta Lev^{*}+\varepsilon$$ (2)

In Formula (2), *Lev* represents the actual capital structure of the enterprise, which is reflected by the asset-liability ratio at the end of the current year; *LagLev* represents the capital structure of the previous year, reflected by the asset-liability ratio at the end of the previous year; Lev* is the target capital structure, *δ* is the speed of capital structure adjustment of the firm, which is measured as the ratio of the actual speed adjustment to the target speed adjustment.

In order to study the impact of non-state-owned shareholder governance on the company ‘s capital structure adjustment speed, we introduce the non-state-owned shareholder governance variables *Mix* (ie, the shareholding ratio of non-state-owned shareholder, the proportion of the directors of the board and the directors, supervisors and top executives appointed by non-state-owned shareholders) based on the model (2), and then substitute the estimated asset-liability ratio from model (1) into model (2) to obtain model (3), which is used to test the impact of non-state-owned shareholder governance on the speed of capital structure adjustment.


$$Lev=\alpha_{0}+(1‐\delta )LagLev+\gamma Mix+\theta Mix\times LagLev+\alpha_{1}Size+\alpha_{2}ROA+\alpha_{3}Fixass+\alpha_{4}Revgro+\alpha_{5}Depre+\alpha_{6}Lev\_med+\alpha_{7}Year+\varepsilon$$ (3)

In Formula (3), *Mix* represents the series of variables that characterize Mixed ownership reform, drawing on Liu et al. [29], we use the dimension to describe mixed ownership reform: equity balance and top executive governance. Equity balance (*Mix*_*S*) is expressed by the sum of the shareholding ratio of the top 10 non-state-owned shareholders (*Nonsoe*_*rat*); top executive governance (*Mix*_*WP*) is expressed by the proportion of the directors of the board (*Mix*_*d*) appointed by the non-state-owned shareholders and the proportion of the directors, supervisors and top executives (*Mix*_*djg*) appointed by the non-state-owned shareholders; *θ*, the coefficient of the interactive item *Mix*×*LagLev*, reflects the impact of the speed of capital structure adjustment. According to H1, our expected *θ* value is significantly negative, indicating that the higher the degree of governance of non-state-owned shareholders, the faster the speed of capital structure adjustment.


$$Dis=\alpha_{0}+\varphi Mix+\alpha_{1}Size+\alpha_{2}ROA+\alpha_{3}Fixass+\alpha_{4}Revgro+\alpha_{5}Depre+\alpha_{6}Lev\_med+\alpha_{7}Year+\varepsilon$$ (4)

*Dis* = |*Lev*−Lev*|, if *Dis* is smaller, the deviation of the capital structure is smaller. If the coefficient *φ* is negative, it reflects that the higher the degree of governance of non-state shareholders, the smaller the deviation between the actual capital structure and the target capital structure. In addition, the model also controls the relevant factors that affect the deviation of the capital structure. The definition of relevant control variables is shown in Table 1.

**Table 1:** Variables and definitions.

Variable	Definition
*Lev*	The asset-liability ratio at the end of the current year.
*Lev* ^*^	Estimated by the model (1).
*Dis*	The absolute value of the actual capital structure and the target capital structure.
*Nonsoe_rat*	The sum of the shareholding ratio of the top 10 non-state-owned shareholders.
*Mix_d*	The proportion of the number of the directors of the board appointed by the non-state-owned shareholders to the total number of the directors of the board of the firm.
*Mix_djg*	The proportion of the number of the directors, supervisors and top executives appointed by the non-state-owned shareholders to the total number of the directors, supervisors and top executives of the firm.
*Size*	The natural logarithm of the asset size at the end of the previous year.
*ROA*	The return on total assets at the end of the previous year.
*Soe_rat*	The sum of the shareholding ratio of the top 10 state-owned shareholders.
*Fixass*	The sum of the inventory and fixed assets at the end of the previous year divided by total assets.
*Revgro*	The growth rate of operating income at the end of last year.
*Depre*	The ratio of fixed asset depreciation and total assets at the end of last year.
*Lev_med*	The median of the capital structure of the enterprise in the same industry in the previous year.

Source: Author’s drawing.

### 3.3 Descriptive statistics

Table 2 reports the descriptive statistics of the main variables. The mean of the actual capital structure (*Lev*) is 0.519, the median is 0.531, and the assets are nearly twice the liabilities, indicating that the debt level of state-owned enterprises is generally reasonable. The mean of the sum of the shareholding ratio of the top 10 non-state-owned shareholders (*Nonsoe*_*rat*) is 11.8%, which, compared with state-owned shareholders, is still a low shareholding ratio; In term of top executive appointment, the proportion of the number of the directors of the board appointed by the non-state-owned shareholders (*Mix*_*d*) is 3.2%, and the proportion of the number of the directors, supervisors and top executives appointed by the non-state-owned shareholders (*Mix*_*djg*) is 1.8%, which are much less than the 11.8% of the average of the *Nonsoe_rat*, indicating the phenomenon that same shareholding companies with different rights exists generally and rights among top executives have not been effectively guaranteed.

**Table 2:** Descriptive statistics.

Variables	Mean	Median	Standard Deviation	Minimum	Maximum
*Lev*	0.519	0.531	0.202	0.084	0.924
*Nonsoe_rat*	0.118	0.120	0.175	0.009	0.924
*Mix_d*	0.032	0	0.361	0	0.364
*Mix_djg*	0.018	0.092	0.549	0	0.279
*Size*	22.564	22.419	1.278	18.160	28.069
*Roa*	0.029	0.027	0.054	-0.585	0.381
*Fixass*	0.432	0.424	0.188	0.003	0.971
*Revgro*	0.154	0.089	0.409	-0.499	2.680
*Depre*	0.166	0.126	0.125	0.001	0.711
*Lev_med*	0.463	0.441	0.124	0.218	0.755
*Observations*	6005				

Source: China Stock Market & Accounting Research Database.

## 4. Empirical results analysis

### 4.1 Regression analysis

#### 4.1.1 The impact of non-state shareholder governance on the speed of recapitalization and the degree of deviation from the capital structure of state-owned enterprises

Table 3 is the regression results of model (3), and it reports the impact of non-state-owned shareholder governance on the speed of capital structure adjustment. In this model, we mainly examine the coefficients of the interaction term (*Mix*×*LagLev*) of the non-state-owned shareholder governance (*Mix*) and capital structure last year (*LagLev*). In column (1), the coefficient interactive item *Nonsoe*_*rat*×*LagLev* is negative but not significant, which means that the ordinary shareholding of non-state-owned shareholders in SOEs is difficult to effectively affect the decision-making behavior on capital structure; Column (2) shows that when the non-state-owned shareholder’s shareholding ratio is greater than or equal to 10%, the coefficient of *Nonsoe*_*rat*×*LagLev* is negatively significant at the level of 1%. This means that when the percentage of shares of non-state-owned shareholders reach to a certain level, they can effectively improve the speed of capital structure adjustment. Columns (3) and (4) are the influences of non-state-owned shareholders’ appointment of directors of board (*Mix*_*d*) and directors, supervisors and top executives (*Mix*_*djg*) on the speed of capital structure adjustment. The coefficients of their interactive item with the firm’ capital structure last year are negatively significant at the 1% and 5% levels, respectively, implying that non-state-owned shareholders appoint senior management to participate in corporate governance in SOEs can effectively accelerate the speed of capital structure adjustment, which is consistent with our assumption.

**Table 3:** The non-state-owned shareholder ‘s governance and the speed of the capital structure adjustment.

*Lev*	(1) Nonsoe_rat	(2) Nonsoe_rat >10%	(3) Mix_d	(4) Mix_djg
*Mix*	0.069** (2.34)	0.023*** (2.85)	0.013*** (2.77)	0.009*** (3.02)
*LagLev*	0.869*** (57.83)	0.858*** (51.00)	0.862*** (52.88)	0.845*** (51.99)
*Mix×LaLev*	-0.059 (-1.46)	-0.072*** (-2.90)	-0.024*** (-3.01)	-0.017** (-2.19)
*Size*	0.012*** (9.77)	0.011*** (7.66)	0.010*** (8.11)	0.011*** (10.79)
*Roa*	-0.593*** (-19.32)	-0.591*** (-13.50)	-0.577*** (-13.01)	-0.593*** (-13.04)
*Fixass*	0.016** (2.31)	0.017*** (3.16)	0.014*** (3.43)	0.016*** (3.42)
*Revgro*	-0.034*** (-7.98)	-0.035*** (-7.54)	-0.168*** (-6.58)	-0.035*** (-6.53)
*Depre*	-0.054*** (-6.87)	-0.054*** (-4.58)	-0.049*** (-8.12)	-0.053*** (-8.14)
*Lev_med*	0.032*** (3.79)	0.032** (2.35)	0.028*** (4.95)	0.035*** (4.93)
*Constant*	-0.165*** (-7.67)	-0.156*** (-6.10)	-0.141*** (-9.06)	-0.154*** (-9.09)
*Year*	controlled	controlled	controlled	controlled
*N*	6005	6005	6005	6005
*Adj_R* ^*2*^	0.886	0.884	0.843	0.885

Note: The t-statistic after Cluster is in parentheses.

***, **, and * represent significance levels of 1%, 5%, and 10%, respectively.

Source: Author’s Calculations

Table 4 reports the regression results of the model (4). It examines the impact of non-state-owned shareholder governance on the deviation of the capital structure. Column (1) reports that the coefficient of shareholding ratio of non-state-owned shareholders (*Nonsoe*_*rat*) is not significant, while in column (2), when the *Nonsoe_rat* is greater than or equal to 10%, the coefficient is significant at 5% level, indicating that when the shareholding ratio of non-state-owned shareholders reaches a certain percentage, they can effectively adjust the deviation of capital structure; Column (3) the coefficients of the non-state-owned shareholders’ appointment of directors of board (*Mix*_*d*) and directors, supervisors and top executives (*Mix*_*djg*) are negatively significant at the 5% level. This means that the greater the proportion of the senior management appointment of non-state-owned shareholders, the smaller the deviation between the actual capital structure and the target capital structure, which is consistent with the assumption. Therefore, the overall results in Tables 3 and 4 support hypothesis 1.

**Table 4:** The non-state-owned shareholder ‘s governance and the degree of deviation of capital structure.

*Dis*	(1) Nonsoe_rat	(2) Nonsoe_rat >10%	(3) Mix_d	(4) Mix_djg
*Mix*	-0.017 (-1.46)	-0.031** (-2.43)	-0.008** (-2.14)	-0.007** (-2.30)
*Controls*	Controlled	Controlled	Controlled	Controlled
*Year*	Controlled	Controlled	Controlled	Controlled
*N*	6005	6005	6005	6005
*Adj_R2*	0.067	0.076	0.112	0.124

Note: The t-statistic after Cluster is in parentheses.

***, **, and * represent significance levels of 1%, 5%, and 10%, respectively.

Source: Author’s Calculations

#### 4.1.2 The influence of non-state-owned shareholder governance on the speed of real capital structure adjustment

Table 5 is the regression results of model (3). This paper further explores the impact of non-state-owned shareholder’s governance on the speed of capital structure adjustment when the actual capital structure is below (under-debt) and above (over-debt) target capital structure. Table 5 shows that in columns (1) and (2), when the debt is insufficient, the coefficient of *Mix*_*d*×*LagLev* and *Mix*_*djg*×*LagLev* is negative, but they are not significant; while in columns (3) and (4), when the company is over-debt, the coefficients of the above two items are negatively significant at the 1% level (and the sample coefficients difference of groups of insufficient debt and excessive debt are respectively significant at the 5% level). This imply that compared to the under-debt samples, among the SOEs which are over-debt, the top executive appointment of non-state-owned shareholders has a more significant impact on the speed of capital structure adjustment, and it will accelerate the downward adjustment of capital structure. This is consistent with our hypothesis 2.

**Table 5:** The non-state-owned shareholder ‘s governance and the speed of the capital structure adjustment.

*Lev*	Under-debt		Over-debt	
	(1) Mix*_d*	(2) Mix*_djg*	(3) Mix*_d*	(4) Mix*_djg*
*Mix*	0.011 (1.53)	0.009 (1.48)	0.037** (2.00)	0.023* (1.76)
*Laglev*	0.644*** (23.58)	0.645*** (23.34)	0.703*** (41.08)	0.702*** (40.99)
*Mix×Laglev*	-0.024 (-1.21)	-0.019 (-1.12)	-0.061*** (-2.94)	-0.041*** (-2.87)
*Controls*	Controlled	Controlled	Controlled	Controlled
*Year*	Controlled	Controlled	Controlled	Controlled
*N*	2980	2980	3025	3025
*Adj_R* ^*2*^	0.839	0.843	0.833	0.833

Note: The t-statistic after Cluster is in parentheses.

***, **, and * represent significance levels of 1%, 5%, and 10%, respectively.

Source: Author’s Calculations

Table 6 is the regression result of model (4), which examines the impact of non-state-owned shareholder’s governance on the degree of deviation of capital structure under insufficient debt and excessive debt situations. In columns (1) and (2), the coefficients of the directors of the board appointed by the non-state-owned shareholders (*Mix*_*d*) and the directors, supervisors and top executives appointed by non-state-owned shareholders (*Mix*_*djg*) are positive, but both are not significant. It implies that in the case of insufficient debt, mixed ownership reform has little effect on the deviation of the actual capital structure from the target capital structure; Columns (3) and (4) presents negatively significant coefficients of *Mix*_*d* and *Mix*_*djg* at the 1% level, which means that in the case of excessive debt, the higher the degree of high-level governance of non-state shareholders, the less the actual capital structure deviates from the target capital structure (the sample coefficients difference of groups of insufficient debt and excessive debt are respectively significant at the 1% level), indicating that non-state-owned shareholder governance has reduced excessive debt and reduced the degree of deviation of the capital structure of over-debt companies. This result is consistent with hypothesis H2. Therefore, the overall regression results in Tables 5 and 6 support the hypothesis H2.

**Table 6:** The non-state-owned shareholder ‘s governance and the degree of deviation of capital structure.

*Dis*	Under-debt		Over-debt	
	(1) Mix*_d*	(2) Mix*_djg*	(3) Mix*_d*	(4) Mix*_djg*
*Mix*	-0.037 (-0.58)	-0.023 (-1.10)	-0.012*** (-2.86)	-0.011*** (-3.25)
*Controls*	Controlled	Controlled	Controlled	Controlled
*Year*	Controlled	Controlled	Controlled	Controlled
*N*	3025	3025	3025	3025
*Adj_R2*	0.833	0.833	0.152	0.153

Note: The t-statistic after Cluster is in parentheses.

***, **, and * represent significance levels of 1%, 5%, and 10%, respectively.

Source: Author’s Calculations

### 4.2 Further analysis

#### 4.2.1 The role of the level of government control

Table 7 is the regression results of the model (3), which reports the impact of mixed ownership reform at different levels of government control on the speed of capital structure adjustment. Columns (1) and (2) shows the coefficients of the interactive items (*Mix*×*Laglev*) of the directors of the board appointed by the non-state-owned shareholders (*Mix*_*d*) and the capital structure in the firm last year (*Laglev*), and the interactive items of the directors, supervisors and top executives appointed by non-state-owned shareholders (*Mix*_*djg*) and *Laglev* in central state-owned enterprises (CSOEs) are negative, but both are not significant. However, these two coefficients in local state-owned enterprises (LSOEs) are negatively significant at 5% level (and the sample coefficients difference of groups of monopolistic state-owned enterprises and competitive state-owned enterprises are respectively significant at the 5% level), indicating that the top executive appointment of non-state-owned shareholders in LSOEs accelerate the adjustment of capital structure, compared to that in CSOEs. This is consistent with our hypothesis 3.

**Table 7:** The role of the level of government control.

Lev	CSOEs		LSOEs		MSOEs		CEs	
	(1) Mix*_d*	(2) Mix*_djg*	(3) Mix*_d*	(4) Mix*_djg*	(5) Mix*_d*	(6) Mix*_djg*	(7) Mix*_d*	(8) Mix*_djg*
*Mix*	0.021* (1.73)	0.028 (1.33)	0.034** (2.45)	0.012** (2.17)	0.031** (2.44)	0.039* (1.68)	0.029*** (2.89)	0.028*** (2.88)
*Laglev*	0.869*** (47.12)	0.872*** (48.79)	0.844*** (49.77)	0.843*** (50.13)	0.734*** (49.18)	0.872*** (50.55)	0.851*** (50.06)	0.849*** (49.84)
*Mix×Laglev*	-0.021 (-1.17)	-0.022 (-1.21)	-0.016** (-2.06)	-0.015** (-2.04)	-0.027 (-1.02)	-0.024 (-1.06)	-0.021** (-2.11)	-0.019** (-2.13)
*Controls*	Controlled	Controlled	Controlled	Controlled	Controlled	Controlled	Controlled	Controlled
*Year*	Controlled	Controlled	Controlled	Controlled	Controlled	Controlled	Controlled	Controlled
*N*	2031	2031	3974	3974	741	741	5264	5264
*Adj_R* ^*2*^	0.863	0.898	0.857	0.887	0.872	0.881	0.867	0.887

Note: The t-statistic after Cluster is in parentheses.

***, **, and * represent significance levels of 1%, 5%, and 10%, respectively.

Source: Author’s Calculations

Table 8 is the regression results of the model (4), which reports the impact of mixed ownership reform of state-owned enterprises at different levels of government control on the deviation of the capital structure. Column (1) and column (2) present that the coefficients of *Mix*_*d* and *Mix*_*djg* in CSOEs are all negative, but not significant, while those in LSOEs in column (3) and column (4) are negatively significant at the 5% level (and the sample coefficients difference of groups of the CSOEs and LSOEs are respectively significant at the 5% level), which indicates that compared with that in central enterprises, the higher the degree of governance of non-state-owned shareholders in local state-owned enterprises, the less their deviation from the target capital structure. This is consistent with hypothesis H3. Based on the above analysis, column (1) and (4) in Tables 7 and 8 generally support hypothesis H3.

**Table 8:** The role of industry competition level.

*Dis*	CSOEs		LSOEs		MSOEs		CEs	
	(1) Mix*_d*	(2) Mix*_djg*	(3) Mix*_d*	(4) Mix*_djg*	(5) Mix*_d*	(6) Mix*_djg*	(7) Mix*_d*	(8) Mix*_djg*
*Mix*	-0.008 (-1.32)	-0.002 (-1.04)	-0.017** (-2.33)	-0.002** (-2.03)	-0.006 (-1.17)	-0.005 (-1.51)	-0.002** (-2.18)	-0.003** (-2.23)
*Controls*	Controlled	Controlled	Controlled	Controlled	Controlled	Controlled	Controlled	Controlled
*Year*	Controlled	Controlled	Controlled	Controlled	Controlled	Controlled	Controlled	Controlled
*N*	2031	2031	2031	2031	741	2031	3974	3974
*Adj_R* ^*2*^	0.176	0.133	0.176	0.133	0.135	0.138	0.146	0.141

Note: The t-statistic after Cluster is in parentheses

***, **, and * represent significance levels of 1%, 5%, and 10%, respectively.

Source: Author’s Calculations

#### 4.2.2 The role of industry competition level

Column (5) and column (8) in Table 7 report the results of non-state-owned shareholder’s governance affecting the speed of capital structure adjustment in different industries. In column (5) and (6), the coefficients of interactive items *Mix*×*Laglev* in monopolistic state-owned enterprises (MSOEs) are negative, but not significant, however, in column (7) and column (8) the coefficients are negatively significant at the 1% level in competitive state-owned enterprises (CEs) (and the sample coefficients difference of groups of monopolistic state-owned enterprises and competitive state-owned enterprises are respectively significant at the 5% level), indicating that the higher the proportion of the director of board and directors, supervisors and top executives appointed by non-state-owned shareholders among competitive state-owned enterprises, the faster the capital structure adjustment. This is consistent with hypothesis 4.

Column (5) and column (8) in Table 8 report the impact of non-state-owned shareholder’s governance on the degree of deviation of capital structure in different industries. Similarly, we find the same results. Compared with that in monopolistic state-owned enterprises, the higher the degree of governance of non-state-owned shareholders in competitive state-owned enterprises, the smaller the degree of deviation from the target capital structure. This is consistent with hypothesis H4. Therefore, based on the above analysis, column (5) and (8) in Tables 7 and 8 generally support hypothesis H4.

### 4.3 Mechanism test

#### 4.3.1 Exclude alternative hypothesis

Compared with private enterprises, state-owned enterprises have soft budget constraints. State-owned enterprises often have advantages in financing, and the mixed-ownership reform of state-owned enterprises which introduces non-state-owned shareholders may strengthen their financing constraints, affecting on the speed of capital structure adjustment and deviation. If the non-state shareholder governance of state-owned enterprises and the strengthening of financial constraints are consistent, it will interfere with the conclusions in this paper, since the financial constraints can also affect the capital structure adjustment. Therefore, in order to exclude the alternative hypothesis of financing constraints, we use Kaplan and Zingales [30] to measure the financing constraints, and group the samples by the median of KZ index, and those that are greater than the median are divided into high groups and less than the median is divided into low groups. If the impact of non-state-owned shareholder governance on capital structure decisions is due to the strengthening of financing constraints, then the capital structure of high financing contraint groups will adjust faster and the actual capital structure will deviate from the target capital structure smaller; Since the opportunistic behavior of management is more likely to exist in the low financing constraint group, if the impact of non-state-owned shareholder’s governance on capital structure adjustment is due to the reduction of managerial opportunistic behavior, such impact is more pronounced in lower financing.

Columns (1)-(4) in Table 9 show that, for the sample of low financing constraints, the high-level appointment by mixed ownership reform has significantly improved the speed of capital structure adjustment and supported the conclusion that non-state shareholder governance increases the speed of capital structure adjustment by reducing the managerial opportunistic behavior. Columns (5)-(8) in Table 9 show that, non-state shareholder governance does not narrow the deviation of capital structure through financing constraints. Therefore, the alternative hypothesis of financing constraints mechanism can be excluded.

**Table 9:** Non-state-owned shareholders governance and decision-making on capital structure under different financing constraints.

	Panel A: Regression results of model (3)				Panel B: Regression results of model (4)			
*Lev*/*Dis*	High financing constraints		Low financing constraints		High financing constraints		Low financing constraints	
	(1) Mix*_d*	(2) Mix*_djg*	(3) Mix*_d*	(4) Mix*_djg*	(5) Mix*_d*	(6) Mix*_djg*	(7) Mix*_d*	(8) Mix*_djg*
*Mix*	0.020* (1.85)	0.015* (1.81)	0.014** (2.22)	0.011** (2.16)	-0.005 (-1.13)	-0.003 (-1.02)	-0.009** (-2.03)	-0.017*** (-2.81)
*Laglev*	0.778*** (43.62)	0.799*** (44.59)	0.552*** (45.86)	0.553*** (45.79)				
*Mix×Laglev*	-0.040 (-1.20)	-0.015 (-0.92)	-0.030** (-2.03)	-0.034** (-2.49)				
*Controls*	Controlled	Controlled	Controlled	Controlled	Controlled	Controlled	Controlled	Controlled
*Year*	Controlled	Controlled	Controlled	Controlled	Controlled	Controlled	Controlled	Controlled
*N*	3082	3082	2023	2023	3082	3082	2023	2023
*Adj_R* ^*2*^	0.473	0.434	0.311	0.307	0.121	0.125	0.160	0.162

Note: The t-statistic after Cluster is in parentheses.

***, **, and * represent significance levels of 1%, 5%, and 10%, respectively.

Source: Author’s Calculations

#### 4.3.2 Mediation test

To further explain whether non-state shareholder governance will reduce the opportunistic behavior of management and affect the decision of capital structure, we follow the intermediary effect test of Baron and Kenny [31] to analyze whether the opportunistic behavior of management is the transmission path for the relationship between the governance of the non-state-owned shareholders and capital structure decision-making. The test results are reported in Table 10.

**Table 10:** Mediation test result.

*Variables*	(1) *Mcost*	(2) *Asscost*	(3) *Lev*	(4) *Lev*	(5) *Dis*	(6) *Dis*
*Mix_d*	-0.009*** (-2.98)	0.037** (2.21)	0.012** (0.065)	0.011* (0.058)	-0.005** (-2.33)	-0.006** (-2.38)
*LagLev*			0.835*** (48.39)	0.858** (49.31)		
*Mix_d × LagLev*			-0.023** (-2.39)	-0.020** (-2.46)		
Mcost			-0.034 (-1.49)		0.084*** (3.02)	
*LagLev × Mcost*			0.077** (2.36)			
*Asscost*				0.021** (2.32)		-0.008* (-1.84)
*LagLev × Asscost*				-0.027*** (-2.98)		
*Controls*	Controlled	Controlled	Controlled	Controlled	Controlled	Controlled
Industry and Year	Controlled	Controlled	Controlled	Controlled	Controlled	Controlled
*Adj—R* ^*2*^	0.121	0.129	0.883	0.886	0.103	0.107
*N*	6005	6005	6005	6005	6005	6005

Note: The t value after Cluster is in parentheses.

***, **, and * represent significance levels of 1%, 5%, and 10%, respectively.

Source: Author’s Calculations

Firstly, the results in Table 10 show that in column (1), the coefficient of the proportion of directors of the board appointed by non-state-owned shareholders (*Mix*_*d*) is -0.009, which is negatively significant at the 1% level, indicating that the higher the proportion of directors of the board appointed by non-state-owned shareholders, the lower the management cost rate. This means that the governance of the non-state-owned shareholder reduces the managerial agent cost; column (2) reports the regression result of the asset turnover rate (*Asscost*), the coefficient is positively significant at 5%. This shows that the higher the proportion of directors of the board appointed by non-state-owned shareholders, the greater the asset turnover rate, which also illustrate that the non-state-owned shareholders’ governance reduces the agency costs.

Secondly, this paper examines the impact of managerial agency costs on capital structure decisions. In column (3), the coefficient of the interactive item (*LagLev*×*Mcost*) is positively significant at 5% level, and in column (4) the coefficient of the interactive item (*LagLev*×*Asscost*) is negatively significant at 5% level. These results show that the management cost rate is negatively correlated with the asset turnover rate, which implies that the lower the agency cost, the faster the adjustment of corporate capital structure. Similarly, column (5) and (6) also prove the same conclusion. The coefficients of *Mcost* and *Asscost* are positively significant at the 1% level, and negatively significant at the 10% level respectively, indicating that the lower the agency cost, the smaller the deviation of the capital structure.

Finally, this paper examines the changes in the relationship between the non-state-owned shareholders’ governance and the capital structure decisions-making. Seen from the Column (3) in Table 10, the agency cost measured by the management cost rate is used as an intermediary. The coefficients of the interactive items (*Mix*_*d*×*LagLev*) is significantly less than 0 at the 5% level.

In Column (3) of Table 10, the absolute value and the significant level of the coefficient *Mix*_*d*×*LagLev* is 0.023, which is less than that in column (4) of Table 3. In Table 10, the agency cost of management’s opportunistic behavior measured by the asset turnover rate is an intermediary in column (4), and the coefficient *Mix*_*d*×*LagLev* is -0.020, which is significantly less than 0 at the 5% level, and its absolute value and its significant level is less than that in column (4) of Table 3. The Z values of SOBEL test are 3.198 and 3.427 respectively, statistically significant at the 1% level, and *θ* in *path c*_1_ is significant and is also less than that in *path a*_1_. The coefficients of *Mix*_*d* in columns (5) and (6) of Table 8 are -0.005 and -0.006, respectively, which are negatively significant at the 5% level, and their absolute values are less than the absolute value of the ratio of directors’ appointment of non-state-owned shareholders, with 0.008. It can be seen that the agency costs characterized by the management cost rate and asset turnover rate play a part of the intermediary role in the impact of non-state-owned shareholders’ governance on capital structure decisions.

### 4.4 Robustness test

#### 4.4.1 Endogenous analysis

First, we deal with endogeneity using 2SLS. Following Liu et al. [29], this paper uses a two-stage method to eliminate the problem of missing variables caused by unobservable factors at the enterprise level. This paper uses the regional annual GDP and the industry average of the proportion of senior executives appointed by non-state-owned shareholders as the instrumental variables that affect the governance level of non-state-owned shareholders in the one-stage regression. The reasons for choosing GDP as an instrument variable is that the more active the regional market economy, the weaker the degree of government intervention, and the higher the degree of mixed ownership reform and non-state-owned shareholder governance. However, since the regional GDP is a macroeconomic factor, this factor has a weaker impact on the internal capital structure of the companies and is also exogenous to the explained variable [32]. Moreover, the industry average of the proportion of senior executives appointed by non-state-owned shareholders is related to explanatory variables and is also exogenous to the explained variables.

In Table 11, the *panel A* is the regression results of the model (3). Columns (1) and (2) report the estimated results of the second-stage appointment of senior executives by non-state-owned shareholders. The coefficients of the interactive items, *Mix*×*LagLev*, are negatively significant at 5% level, indicating that mixed ownership reform has increased the speed of capital structure adjustment. *Panel B* is the regression result of the model (4). The coefficients *Mix*_*d* and *Mix*_*djg* in columns (5) and (6) are negatively significant at the level of 1%. This means that the proportion of senior executives appointed by non-state-owned shareholders narrows the deviation of the capital structure. After using the two-stage endogenous analysis, it was found that the influence of non-state-owned shareholders’ governance on the dynamic adjustment of capital structure still exists.

**Table 11:** Robustness test.

*Variables*	Panel A:Regression results of model (3)				Panel B:Regression results of model (4)			
	2SLS	2SLS	Heckman	ILev	2SLS		Heckman	ILev
	(1) Mix*_d*	(2) Mix*_djg*	(3) Mix*_dum*	(4) Mix*_d*	(5) Mix*_d*	(6) Mix*_djg*	(7) Mix*_dum*	(8) Mix*_d*
*Mix*	-0.139* (-1.83)	-0.145* (-1.86)	0.036*** (2.75)	0.007** (2.42)	-0.009*** (-3.13)	-0.008*** (-3.08)	-0.012** (-2.41)	-0.016** (-2.44)
*LagLev*	0.815*** (51.03)	0.818*** (51.06)	0.846*** (49.82)	0.868*** (50.35)				
*Mix* × *LagLev*	-0.039** (-2.23)	-0.042** (-2.28)	-0.064** (-2.16)	-0.034*** (-2.84)				
*Lambda*			0.248 (0.620)				-0.439 (-1.21)	
*Controls*	Controlled	Controlled	Controlled	Controlled	Controlled	Controlled	Controlled	Controlled
*Year*	Controlled	Controlled	Controlled	Controlled	Controlled	Controlled	Controlled	Controlled
*N*	6005	6005	6005	6005	6005	6005	6005	6005
*Adj_R2*	0.867	0.872	0.311	0.307	0.121	0.125	0.872	0.393
*Hansen-test*	P = 0.464	P = 0.459			P = 0.396	P = 0.383		

Note: The t-statistic after Cluster is in parentheses

***, **, and * represent significance levels of 1%, 5%, and 10%, respectively.

Source: Author’s Calculations

Second, the relationship between mixed ownership reform and the dynamic adjustment of capital structure will be disturbed by the endogenous self-selection effect. This paper uses two stages of testing to alleviate the self-selection problem. In Panel A, Column (3) reports that whether the non-state-owned shareholder appoints the directors (*Mix*_*dum*) and its interactive item with *LagLev*. The coefficient of *Mix*×*LagLev* is negatively significant at the 5% level, and the coefficient of *Lambda* is not significant, which implies that non-state-owned shareholders’ governance has improved the speed of the capital structure adjustment. In Panel B, Column (7) shows that the coefficient of *Mix*_*dum* is negatively significant at the 5% level, and the coefficient of the *Lambda* is not significant, indicating that mixed ownership reform reduces the deviation of the actual capital structure and target capital structure. Therefore, the test results are used to further support the original hypothesis.

#### 4.4.2 Variable substitution test

In this paper, the asset-liability ratio (*ILev*) expressed by interest-bearing liabilities is used as the dependent variable, and conduct regression on the model (3) and model (4) again. The regression results are shown in Panel A column (4) and Panel B column (8) in Table 11. Regression result of model (3) in column (4), the coefficient of *Mix*×*LagLev* is negatively significant at the level of 1%. While in Panel B, the coefficient of the proportion of directors appointed by the non-state-owned shareholders (*Mix*_*d*) is negatively significant at the 5% level. Therefore, when the interest-bearing debt ratio is used as a substitute variable for the asset-liability ratio, we make the same conclusion that mixed ownership reform accelerates the adjustment of capital structure and reduces the degree of deviation of the capital structure.

## 5. Conclusion and implication

### 5.1 Conclusion

This paper mainly studies how the non-state-owned shareholder governance affects the capital structure decision-making from the governance dimension of the mixed ownership reform by using data from A-share state-owned listed companies during 2008 to 2021. The study finds that, firstly, the top executives appointment by non-state-owned shareholders can accelerate the speed of capital structure adjustment and reduce the deviation of capital structure, and the above effects work only when the shareholding ratio of non-state-owned shareholders reaches to a certain level. Secondly, when the actual capital structure is higher than the target capital structure, mixed ownership reform will accelerate the downward adjustment of the capital structure and reduce the deviation of the actual capital structure of the over-debt enterprise from the target capital structure. Thirdly, according to the level of government control and the degree of competition in different industries, we find out that the impact of the top executive appointment by non-state-owned shareholders on capital structure decisions is more significant in local state-owned enterprises and competitive state-owned enterprises. In addition, endogenous test and variable substitution test further support the above conclusions. Finally, through the path test, it is found that the effect of non-state-owned shareholders’ governance works not by financing constraints, but by reducing the opportunistic behavior of management, thereby increasing the speed of capital structure adjustment and reducing the deviation of capital structure. This means that the management opportunism or agency cost plays an intermediary role in the effect of non-state-owned shareholders’ decision-making on capital structure.

### 5.2 Policy implication

Based on the above research, we get the following policy implications: First, the mixed ownership reform by continuously introducing heterogeneous shareholders to weaken the opportunistic behavior of state-owned enterprises and improve corporate governance. And it is the key of mixed ownership reform of state-owned enterprises to introduce non-state-owned shareholders to effectively supervise and restrict management, which can reduce the opportunistic behavior of management and improve the quality of corporate governance. Meanwhile, it is also necessary to improve the level of financial literacy and attention of management [33, 34]. Second, the mixed ownership reform should not only mix the shareholding elements but should also mix the actual rights from all elements in shareholders. Allowing non-state-owned shareholders to participate in the governance of state-owned enterprises is conducive to the scientific and rational financial decision-making of the company. Third, the mixed-ownership reform among central state-owned enterprises and original state-owned enterprises in monopolistic industries should be strengthened, and give play to the governance role of non-state shareholders in these types of enterprises, so that the mixed-ownership reform can play a real role in improving the performance of operations and optimizing the quality of decision-making in these enterprises [35].
